# Di-μ-iodido-bis­{[dicyclo­hexyl(phen­yl)phosphine-κ*P*](pyridine-κ*N*)silver(I)}

**DOI:** 10.1107/S160053680901099X

**Published:** 2009-03-31

**Authors:** Bernard Omondi, Reinout Meijboom

**Affiliations:** a Department of Chemistry, University of Johannesburg, PO Box 524, Auckland Park, Johannesburg 2006, South Africa

## Abstract

The title compound, [Ag_2_I_2_(C_5_H_5_N)_2_(C_18_H_27_P)_2_], contains centrosymmetric dinuclear species in which each Ag atom is surrounded by a phosphine ligand, a weakly coordinating pyridine ligand and two iodide anions in a distorted tetra­hedral coordination. The two iodide anions bridge the Ag atoms, which are separated by a distance of 3.1008 (6) Å. The Ag—P distance is 2.4436 (8) Å, Ag—N is 2.386 (3)Å and the Ag—I distances are 2.8186 (4) and 2.9449 (5) Å.

## Related literature

For a review of the chemistry of silver(I) complexes, see: Meijboom *et al.* (2009 ▶). For the coordination chemistry of Ag*X* salts (*X* ^−^ = F^−^, Cl^−^, Br^−^, I^−^, BF_4_^−^, PF_6_^−^, NO_3_^−^ *etc*) with group 15 donor ligands, with the main focus on tertiary phosphines and in their context as potential anti­tumor agents, see: Berners-Price *et al.* (1998 ▶); Liu *et al.* (2008 ▶). For tertiary phosphine silver(I) complexes of mixed-base species, see: Engelhardt *et al.* (1989 ▶); Gotsis *et al.* (1989 ▶); Meijboom & Muller (2006 ▶). The unsymmetrical core (Ag—I—Ag′—I′) may be attributed to the partial separation of dimer into monomer of such complexes, see: Bowmaker *et al.* (1996 ▶); Meijboom & Muller (2006 ▶). For the solution behaviour of [*L*_*n*_Ag*X*] complexes, see: Muetterties & Alegranti (1972 ▶).
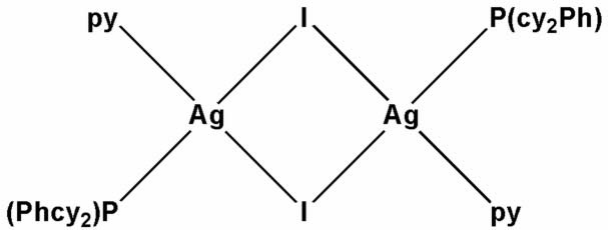


## Experimental

### 

#### Crystal data

[Ag_2_I_2_(C_5_H_5_N)_2_(C_18_H_27_P)_2_]
*M* *_r_* = 1176.47
Triclinic, 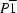

*a* = 9.5970 (12) Å
*b* = 9.9816 (13) Å
*c* = 14.1437 (18) Å
α = 90.484 (3)°
β = 102.404 (2)°
γ = 112.704 (2)°
*V* = 1214.4 (3) Å^3^
*Z* = 1
Mo *K*α radiation
μ = 2.18 mm^−1^
*T* = 293 K
0.3 × 0.22 × 0.09 mm

#### Data collection

Bruker SMART CCD area-detector diffractometer
Absorption correction: multi-scan (*SADABS*; Bruker, 2004 ▶) *T*_min_ = 0.562, *T*_max_ = 0.828
7951 measured reflections
5723 independent reflections
4310 reflections with *I* > 2σ(*I*)
*R*_int_ = 0.014

#### Refinement

*R*[*F* ^2^ > 2σ(*F* ^2^)] = 0.031
*wR*(*F* ^2^) = 0.073
*S* = 1.02
5723 reflections
244 parameters
H-atom parameters constrained
Δρ_max_ = 0.50 e Å^−3^
Δρ_min_ = −0.81 e Å^−3^

### 

Data collection: *SMART* (Bruker, 2004 ▶); cell refinement: *SAINT* (Bruker, 2004 ▶); data reduction: *SAINT*; program(s) used to solve structure: *SHELXS97* (Sheldrick, 2008 ▶); program(s) used to refine structure: *SHELXL97* (Sheldrick, 2008 ▶); molecular graphics: *DIAMOND* (Brandenburg & Putz, 2005 ▶); software used to prepare material for publication: *WinGX* (Farrugia, 1999 ▶).

## Supplementary Material

Crystal structure: contains datablocks global, I. DOI: 10.1107/S160053680901099X/hg2494sup1.cif

Structure factors: contains datablocks I. DOI: 10.1107/S160053680901099X/hg2494Isup2.hkl

Additional supplementary materials:  crystallographic information; 3D view; checkCIF report

## Figures and Tables

**Table 1:** Comparison of geometric parameters (Å, °) for selected [*X*Ag(py)(P_3_)_2_] (*X* = Cl, Br or I) entities

*X*	Ag—*X*	Ag—*X*	Ag⋯Ag	Ag—N	Ag—P	*X*—Ag—*X*	Ag—I—Ag
I^*a*^	2.8186 (4)	2.9449 (5)	3.1008 (6)	2.386 (3)	2.4436 (8)	114.947 (10)	65.053 (10)
I^*b*^	2.8402 (12)	2.8644 (8)	3.1130 (18)	2.392 (3)	2.4489 (12)	113.84 (4)	66.16 (4)
I^*c*^	2.814	2.875	3.343	2.422	2.440	108.02	71.98
Br^*c*^	2.701	2.733	3.499	2.391	2.415	99.85	80.15
Cl^*c*^	2.614	2.618	3.507	2.402	2.400	95.82	84.18

Notes: (*a*) This work; (*b*) Meijboom & Muller (2006 ▶); (*c*) Gotsis *et al.* (1989 ▶), extracted from the Cambridge Structural Database (Allen (2002 ▶), CSD CODES are VEFRUT for *X* = I, VEFRON for *X* = Br and VEFRIH for *X* = Cl.

**Fig. 1.**
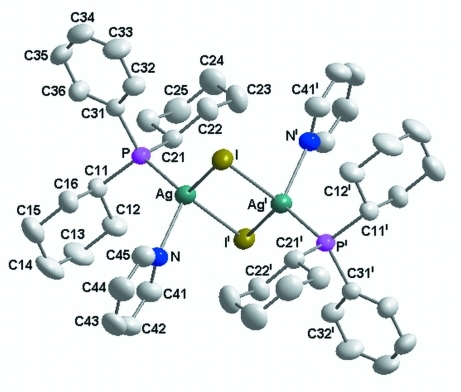
The molecular structure of (I), showing 50% probability displacement ellipsoids. H atoms have been omitted for clarity. For the C atoms, the first digit indicates the ring number and the second digit indicates the position of the atom in the ring. Primed atoms are generated by the symmetry code (1 - x, 1 - y, 1 - z).

## supplementary crystallographic information

### Comment

The chemistry of silver(I) complexes has been reviewed recently with regards to the coordination chemistry, the design of coordination networks and polymers containing nitrogen-donor ligands and to the chemistry of silver scorpionates and carboxylates (Meijboom *et al.*, 2009). Our interest has been on the coordination chemistry of AgX salts (*X*^-^ = F^-^, Cl^-^, Br^-^, I^-^, BF_4_^-^, PF_6_^-^, NO_3_^-^*etc*.) with Group 15 donor ligands with the main focus on tertiary phosphines and in their context as potential antitumor agents (Berners-Price *et al.*, 1998; Liu *et al.*, 2008).

Tertiary phosphine silver(I) complexes of mixed-base species have been reported but are not very common (Meijboom *et al.*, 2009). Examples of these complexes include [XAg(py)(PPh_3_)_2_] (*X* = Cl or Br) (Engelhardt *et al.*, 1989), [XAg(py)PPh_3_]_2_.C_5_H_5_N (*X* = Cl, Br or I) (Gotsis *et al.*, 1989) and [IAg(py)(P-*p*-tol-Ph_3_)]_2_ (Meijboom & Muller, 2006). The preparation of [IAg(py)(Pcy_2_Ph)]_2_ (I) is similar to those reported and involves heating together stoichiometric mixtures of silver(I)iodide and dicyclohexylphenylphosphine in pyridine solution.

As pointed out earlier by Meijboom & Muller (2006), the resulting complex comprises of a 1:1:1 µ,µ'-diiodo-bridged dimer. The Ag atoms of this centrosymmetric title compound are coordinated to a phosphine ligand, a pyridine ligand and two iodide anions in a distorted tetrahedral manner. The bond angles around the Ag atoms are listed in Table 1. The Ag—P, Ag—N and Ag—I bond distances are typical of similar complexes. However the difference in the Ag—I and Ag—I' bond distances [2.8186 (4) and 2.9449 (5) Å] which results in an unsymmetrical core (Ag—I—Ag'-I') of the complex has been attributed to the partial separation of dimer into monomer of such complexes (Bowmaker *et al.*, 1996; Meijboom & Muller, 2006).

In comparison (see Table 2), the same Ag—*X* bond distance seems larger in (I) as compared to those in [XAg(py)(PPh_3_)]_2_.C_5_H_5_N (*X* = Cl, Br or I) (Gotsis *et al.*, 1989) and [IAg(py)(P-(*p*-tol)_3_)]_2_ (Meijboom and Muller, 2006) which are only slightly different. The bond angles in the core (Ag—*X*—Ag' and *X*—Ag—*X*') are similar in (I), [IAg(py)(P-(*p*-tol)_3_)]_2_ and [XAg(py)(PPh_3_)]_2_.C_5_H_5_N (*X* = I). In these structures the Ag—*X*—Ag' is much smaller than *X*—Ag—*X*'. The situation is slightly different for [XAg(py)(PPh_3_)]_2_.C_5_H_5_N (*X* = Cl or Br) in which the two angles are closer to 90°. Similarly the Ag···Ag bond distances are shorter in (I) and [IAg(py)(P-(*p*-tol)_3_)]_2_ but increases in [XAg(py)(PPh_3_)]_2_.C_5_H_5_N (*X* = Cl, Br or I). Ag—P and Ag—N bond distances are comparable in all five structures listed in Table 2.

Despite the number of structural reports of [*L*_n_AgX] complexes, their solution behaviour, initiated by Muetterties & Alegranti (1972), has always shown that the coordinating ligands were labile in all complexes studied. Rapid ligand-exchange reactions have been reported for all ^31^P NMR spectroscopic investigations of ionic Ag^I^ monodentate phosphine complexes, thus making NMR spectroscopy of limited use for these types of complexes.

### Experimental

Silver iodide (0.130 g, 0.43 mmol) and dicyclohexylphenylphosphine (1.009 g, 0.86 mmol) were suspended in pyridine (5 ml). The mixture was heated to give a clear solution. Colourless crystals of the title compound suitable for X-ray crystallography were obtained by slow evaporation.

### Refinement

All hydrogen atoms were positioned geometrically, with C—H = 0.97 Å, and allowed to ride on their parent atoms with *U*_iso_(H) = 1.2*U*_eq_(C).

### Crystal data

**Table:**

[Ag_2_I_2_(C_5_H_5_N)_2_(C_18_H_27_P)_2_]	*Z* = 1
*M**_r_* = 1176.47	*F*(000) = 584
Triclinic, *P*1	*D*_x_ = 1.609 Mg m^−^^3^
Hall symbol: -P 1	Mo *K*α radiation, λ = 0.71073 Å
*a* = 9.5970 (12) Å	Cell parameters from 8087 reflections
*b* = 9.9816 (13) Å	θ = 1.5–28°
*c* = 14.1437 (18) Å	µ = 2.17 mm^−^^1^
α = 90.484 (3)°	*T* = 293 K
β = 102.404 (2)°	Plate, colourless
γ = 112.704 (2)°	0.3 × 0.22 × 0.09 mm
*V* = 1214.4 (3) Å^3^	

### Data collection

**Table:**

Bruker SMART CCD area-detector diffractometer	4310 reflections with *I* > 2σ(*I*)
ω scans	*R*_int_ = 0.014
Absorption correction: multi-scan (*SADABS*; Bruker, 2004)	θ_max_ = 28°, θ_min_ = 1.5°
*T*_min_ = 0.562, *T*_max_ = 0.828	*h* = −12→12
7951 measured reflections	*k* = −11→13
5723 independent reflections	*l* = −14→18

### Refinement

**Table:**

Refinement on *F*^2^	0 restraints
Least-squares matrix: full	H-atom parameters constrained
*R*[*F*^2^ > 2σ(*F*^2^)] = 0.031	*w* = 1/[σ^2^(*F*_o_^2^) + (0.0363*P*)^2^] where *P* = (*F*_o_^2^ + 2*F*_c_^2^)/3
*wR*(*F*^2^) = 0.073	(Δ/σ)_max_ = 0.002
*S* = 1.01	Δρ_max_ = 0.50 e Å^−^^3^
5723 reflections	Δρ_min_ = −0.81 e Å^−^^3^
244 parameters	

### Special details

**Table:**

Geometry. All e.s.d.'s (except the e.s.d. in the dihedral angle between two l.s. planes) are estimated using the full covariance matrix. The cell e.s.d.'s are taken into account individually in the estimation of e.s.d.'s in distances, angles and torsion angles; correlations between e.s.d.'s in cell parameters are only used when they are defined by crystal symmetry. An approximate (isotropic) treatment of cell e.s.d.'s is used for estimating e.s.d.'s involving l.s. planes.

### Fractional atomic coordinates and isotropic or equivalent isotropic displacement parameters (Å^2^)

**Table:**

	*x*	*y*	*z*	*U*_iso_*/*U*_eq_	
Ag	0.64528 (3)	0.51283 (3)	0.579194 (16)	0.04387 (8)	
I	0.67657 (2)	0.67819 (2)	0.418646 (15)	0.04704 (8)	
P	0.75790 (9)	0.61782 (8)	0.74849 (5)	0.03498 (17)	
N	0.7116 (3)	0.3242 (3)	0.5233 (2)	0.0496 (7)	
C11	0.8282 (4)	0.4979 (3)	0.8258 (2)	0.0420 (7)	
H11	0.8702	0.545	0.8926	0.05*	
C12	0.6951 (4)	0.3510 (4)	0.8252 (3)	0.0545 (9)	
H12A	0.6467	0.3071	0.7587	0.065*	
H12B	0.6171	0.366	0.8525	0.065*	
C13	0.7541 (6)	0.2473 (5)	0.8845 (3)	0.0758 (12)	
H13A	0.792	0.2863	0.9524	0.091*	
H13B	0.6687	0.1531	0.8799	0.091*	
C14	0.8837 (6)	0.2275 (5)	0.8483 (3)	0.0793 (13)	
H14A	0.9225	0.1663	0.8897	0.095*	
H14B	0.8427	0.1782	0.7828	0.095*	
C15	1.0152 (5)	0.3712 (5)	0.8482 (3)	0.0745 (12)	
H15A	1.0921	0.3552	0.8204	0.089*	
H15B	1.0647	0.415	0.9147	0.089*	
C16	0.9582 (4)	0.4757 (4)	0.7898 (3)	0.0535 (9)	
H16A	0.9202	0.4372	0.7218	0.064*	
H16B	1.0446	0.5692	0.7948	0.064*	
C21	0.6083 (4)	0.6419 (3)	0.8017 (2)	0.0403 (7)	
H21	0.5195	0.5473	0.7889	0.048*	
C22	0.5502 (4)	0.7498 (4)	0.7480 (2)	0.0523 (8)	
H22A	0.5176	0.7192	0.6787	0.063*	
H22B	0.6344	0.8457	0.7585	0.063*	
C23	0.4137 (5)	0.7582 (5)	0.7837 (3)	0.0680 (11)	
H23A	0.3842	0.8322	0.752	0.082*	
H23B	0.3253	0.6653	0.7659	0.082*	
C24	0.4547 (5)	0.7946 (5)	0.8927 (3)	0.0713 (11)	
H24A	0.5336	0.8932	0.9096	0.086*	
H24B	0.3633	0.7912	0.9132	0.086*	
C25	0.5139 (5)	0.6903 (5)	0.9458 (3)	0.0643 (10)	
H25A	0.4306	0.5939	0.9355	0.077*	
H25B	0.5458	0.7213	1.015	0.077*	
C26	0.6511 (4)	0.6823 (4)	0.9116 (2)	0.0512 (8)	
H26A	0.6819	0.6099	0.9447	0.061*	
H26B	0.7386	0.776	0.9282	0.061*	
C31	0.9209 (3)	0.7958 (3)	0.7758 (2)	0.0386 (7)	
C32	0.9355 (4)	0.8901 (4)	0.7032 (2)	0.0473 (8)	
H32	0.8637	0.8606	0.6435	0.057*	
C33	1.0555 (5)	1.0272 (4)	0.7185 (3)	0.0628 (10)	
H33	1.0619	1.0899	0.6698	0.075*	
C34	1.1638 (5)	1.0704 (4)	0.8043 (4)	0.0713 (12)	
H34	1.2464	1.1611	0.8134	0.086*	
C35	1.1515 (5)	0.9803 (4)	0.8779 (3)	0.0714 (12)	
H35	1.2242	1.011	0.9374	0.086*	
C36	1.0303 (4)	0.8434 (4)	0.8631 (3)	0.0583 (9)	
H36	1.0227	0.7828	0.913	0.07*	
C41	0.6644 (5)	0.1923 (4)	0.5544 (3)	0.0605 (10)	
H41	0.592	0.1694	0.5925	0.073*	
C42	0.7168 (5)	0.0881 (4)	0.5333 (3)	0.0694 (11)	
H42	0.6801	−0.0032	0.5562	0.083*	
C43	0.8250 (5)	0.1217 (5)	0.4776 (3)	0.0759 (12)	
H43	0.8629	0.0536	0.462	0.091*	
C44	0.8749 (5)	0.2560 (5)	0.4461 (3)	0.0742 (12)	
H44	0.9491	0.2818	0.4092	0.089*	
C45	0.8155 (4)	0.3540 (4)	0.4688 (3)	0.0577 (9)	
H45	0.8491	0.445	0.4453	0.069*	

### Atomic displacement parameters (Å^2^)

**Table:**

	*U*^11^	*U*^22^	*U*^33^	*U*^12^	*U*^13^	*U*^23^
Ag	0.05080 (15)	0.04133 (14)	0.03791 (14)	0.01641 (11)	0.01097 (11)	0.00158 (10)
I	0.04314 (13)	0.04213 (13)	0.04845 (13)	0.00716 (9)	0.01405 (9)	0.01362 (9)
P	0.0372 (4)	0.0322 (4)	0.0333 (4)	0.0111 (3)	0.0089 (3)	0.0039 (3)
N	0.0534 (17)	0.0422 (16)	0.0540 (17)	0.0210 (13)	0.0105 (13)	−0.0023 (13)
C11	0.0486 (18)	0.0421 (18)	0.0354 (16)	0.0202 (15)	0.0056 (13)	0.0075 (13)
C12	0.069 (2)	0.044 (2)	0.058 (2)	0.0240 (18)	0.0271 (18)	0.0196 (16)
C13	0.113 (4)	0.055 (2)	0.077 (3)	0.043 (3)	0.039 (3)	0.033 (2)
C14	0.116 (4)	0.065 (3)	0.083 (3)	0.061 (3)	0.029 (3)	0.025 (2)
C15	0.087 (3)	0.082 (3)	0.073 (3)	0.059 (3)	0.008 (2)	0.010 (2)
C16	0.049 (2)	0.056 (2)	0.060 (2)	0.0265 (18)	0.0120 (16)	0.0077 (17)
C21	0.0408 (16)	0.0372 (17)	0.0412 (17)	0.0113 (14)	0.0144 (13)	0.0015 (13)
C22	0.058 (2)	0.061 (2)	0.048 (2)	0.0319 (18)	0.0179 (16)	0.0101 (16)
C23	0.061 (2)	0.089 (3)	0.069 (3)	0.045 (2)	0.018 (2)	0.012 (2)
C24	0.072 (3)	0.083 (3)	0.077 (3)	0.041 (2)	0.036 (2)	0.006 (2)
C25	0.070 (3)	0.074 (3)	0.052 (2)	0.023 (2)	0.0297 (19)	0.0040 (19)
C26	0.060 (2)	0.058 (2)	0.0408 (18)	0.0264 (18)	0.0168 (16)	0.0084 (16)
C31	0.0359 (16)	0.0343 (16)	0.0443 (17)	0.0119 (13)	0.0106 (13)	0.0023 (13)
C32	0.0523 (19)	0.0419 (18)	0.0453 (18)	0.0133 (15)	0.0170 (15)	0.0050 (14)
C33	0.065 (2)	0.044 (2)	0.071 (3)	0.0054 (18)	0.029 (2)	0.0085 (18)
C34	0.051 (2)	0.041 (2)	0.107 (4)	0.0028 (17)	0.019 (2)	−0.005 (2)
C35	0.058 (2)	0.049 (2)	0.081 (3)	0.0091 (19)	−0.014 (2)	−0.013 (2)
C36	0.057 (2)	0.049 (2)	0.055 (2)	0.0164 (18)	−0.0032 (17)	0.0035 (17)
C41	0.066 (2)	0.050 (2)	0.069 (2)	0.0227 (19)	0.023 (2)	0.0046 (18)
C42	0.083 (3)	0.046 (2)	0.080 (3)	0.030 (2)	0.013 (2)	0.003 (2)
C43	0.080 (3)	0.068 (3)	0.094 (3)	0.050 (3)	0.012 (3)	−0.009 (2)
C44	0.066 (3)	0.071 (3)	0.097 (3)	0.035 (2)	0.029 (2)	−0.003 (2)
C45	0.053 (2)	0.052 (2)	0.069 (2)	0.0200 (18)	0.0172 (18)	0.0013 (18)

### Geometric parameters (Å, °)

**Table:**

Ag—N	2.386 (3)	C22—H22B	0.97
Ag—P	2.4436 (8)	C23—C24	1.510 (5)
Ag—I	2.8186 (4)	C23—H23A	0.97
Ag—I^i^	2.9449 (5)	C23—H23B	0.97
Ag—Ag^i^	3.1008 (6)	C24—C25	1.503 (5)
I—Ag^i^	2.9449 (4)	C24—H24A	0.97
P—C31	1.827 (3)	C24—H24B	0.97
P—C11	1.847 (3)	C25—C26	1.525 (5)
P—C21	1.847 (3)	C25—H25A	0.97
N—C41	1.329 (4)	C25—H25B	0.97
N—C45	1.334 (4)	C26—H26A	0.97
C11—C12	1.527 (5)	C26—H26B	0.97
C11—C16	1.532 (4)	C31—C36	1.379 (4)
C11—H11	0.98	C31—C32	1.391 (4)
C12—C13	1.536 (5)	C32—C33	1.384 (5)
C12—H12A	0.97	C32—H32	0.93
C12—H12B	0.97	C33—C34	1.358 (6)
C13—C14	1.521 (6)	C33—H33	0.93
C13—H13A	0.97	C34—C35	1.376 (6)
C13—H13B	0.97	C34—H34	0.93
C14—C15	1.501 (6)	C35—C36	1.389 (5)
C14—H14A	0.97	C35—H35	0.93
C14—H14B	0.97	C36—H36	0.93
C15—C16	1.526 (5)	C41—C42	1.374 (5)
C15—H15A	0.97	C41—H41	0.93
C15—H15B	0.97	C42—C43	1.378 (6)
C16—H16A	0.97	C42—H42	0.93
C16—H16B	0.97	C43—C44	1.353 (6)
C21—C26	1.528 (4)	C43—H43	0.93
C21—C22	1.530 (4)	C44—C45	1.376 (5)
C21—H21	0.98	C44—H44	0.93
C22—C23	1.532 (5)	C45—H45	0.93
C22—H22A	0.97		
			
N—Ag—P	118.15 (7)	C21—C22—H22A	109.4
N—Ag—I	98.31 (7)	C23—C22—H22A	109.4
P—Ag—I	123.82 (2)	C21—C22—H22B	109.4
N—Ag—I^i^	95.85 (7)	C23—C22—H22B	109.4
P—Ag—I^i^	102.83 (2)	H22A—C22—H22B	108
I—Ag—I^i^	114.947 (10)	C24—C23—C22	111.6 (3)
N—Ag—Ag^i^	103.19 (7)	C24—C23—H23A	109.3
P—Ag—Ag^i^	135.80 (2)	C22—C23—H23A	109.3
I—Ag—Ag^i^	59.443 (10)	C24—C23—H23B	109.3
I^i^—Ag—Ag^i^	55.505 (11)	C22—C23—H23B	109.3
Ag—I—Ag^i^	65.053 (10)	H23A—C23—H23B	108
C31—P—C11	104.16 (14)	C25—C24—C23	111.7 (3)
C31—P—C21	104.32 (14)	C25—C24—H24A	109.3
C11—P—C21	105.76 (14)	C23—C24—H24A	109.3
C31—P—Ag	119.07 (10)	C25—C24—H24B	109.3
C11—P—Ag	112.57 (10)	C23—C24—H24B	109.3
C21—P—Ag	109.89 (10)	H24A—C24—H24B	107.9
C41—N—C45	116.9 (3)	C24—C25—C26	111.9 (3)
C41—N—Ag	122.4 (2)	C24—C25—H25A	109.2
C45—N—Ag	120.1 (2)	C26—C25—H25A	109.2
C12—C11—C16	110.3 (3)	C24—C25—H25B	109.2
C12—C11—P	110.5 (2)	C26—C25—H25B	109.2
C16—C11—P	109.9 (2)	H25A—C25—H25B	107.9
C12—C11—H11	108.7	C25—C26—C21	110.9 (3)
C16—C11—H11	108.7	C25—C26—H26A	109.5
P—C11—H11	108.7	C21—C26—H26A	109.5
C11—C12—C13	110.9 (3)	C25—C26—H26B	109.5
C11—C12—H12A	109.5	C21—C26—H26B	109.5
C13—C12—H12A	109.5	H26A—C26—H26B	108.1
C11—C12—H12B	109.5	C36—C31—C32	117.7 (3)
C13—C12—H12B	109.5	C36—C31—P	124.7 (3)
H12A—C12—H12B	108	C32—C31—P	117.6 (2)
C14—C13—C12	111.5 (3)	C33—C32—C31	121.0 (3)
C14—C13—H13A	109.3	C33—C32—H32	119.5
C12—C13—H13A	109.3	C31—C32—H32	119.5
C14—C13—H13B	109.3	C34—C33—C32	120.2 (4)
C12—C13—H13B	109.3	C34—C33—H33	119.9
H13A—C13—H13B	108	C32—C33—H33	119.9
C15—C14—C13	111.6 (4)	C33—C34—C35	120.1 (3)
C15—C14—H14A	109.3	C33—C34—H34	119.9
C13—C14—H14A	109.3	C35—C34—H34	119.9
C15—C14—H14B	109.3	C34—C35—C36	119.7 (4)
C13—C14—H14B	109.3	C34—C35—H35	120.1
H14A—C14—H14B	108	C36—C35—H35	120.1
C14—C15—C16	111.4 (4)	C31—C36—C35	121.2 (4)
C14—C15—H15A	109.4	C31—C36—H36	119.4
C16—C15—H15A	109.4	C35—C36—H36	119.4
C14—C15—H15B	109.4	N—C41—C42	123.5 (4)
C16—C15—H15B	109.4	N—C41—H41	118.2
H15A—C15—H15B	108	C42—C41—H41	118.2
C15—C16—C11	112.1 (3)	C41—C42—C43	118.6 (4)
C15—C16—H16A	109.2	C41—C42—H42	120.7
C11—C16—H16A	109.2	C43—C42—H42	120.7
C15—C16—H16B	109.2	C44—C43—C42	118.5 (4)
C11—C16—H16B	109.2	C44—C43—H43	120.8
H16A—C16—H16B	107.9	C42—C43—H43	120.8
C26—C21—C22	110.5 (3)	C43—C44—C45	119.7 (4)
C26—C21—P	116.8 (2)	C43—C44—H44	120.2
C22—C21—P	110.4 (2)	C45—C44—H44	120.2
C26—C21—H21	106.2	N—C45—C44	122.8 (4)
C22—C21—H21	106.2	N—C45—H45	118.6
P—C21—H21	106.2	C44—C45—H45	118.6
C21—C22—C23	111.1 (3)		
			
N—Ag—I—Ag^i^	100.46 (7)	C31—P—C21—C26	−61.2 (3)
P—Ag—I—Ag^i^	−127.34 (3)	C11—P—C21—C26	48.3 (3)
I^i^—Ag—I—Ag^i^	0	Ag—P—C21—C26	170.1 (2)
N—Ag—P—C31	102.47 (14)	C31—P—C21—C22	66.0 (3)
I—Ag—P—C31	−21.28 (12)	C11—P—C21—C22	175.6 (2)
I^i^—Ag—P—C31	−153.61 (11)	Ag—P—C21—C22	−62.7 (2)
Ag^i^—Ag—P—C31	−100.40 (12)	C26—C21—C22—C23	−55.6 (4)
N—Ag—P—C11	−19.81 (14)	P—C21—C22—C23	173.7 (3)
I—Ag—P—C11	−143.57 (11)	C21—C22—C23—C24	54.9 (5)
I^i^—Ag—P—C11	84.11 (11)	C22—C23—C24—C25	−54.4 (5)
Ag^i^—Ag—P—C11	137.32 (11)	C23—C24—C25—C26	54.9 (5)
N—Ag—P—C21	−137.39 (13)	C24—C25—C26—C21	−55.8 (4)
I—Ag—P—C21	98.85 (11)	C22—C21—C26—C25	55.9 (4)
I^i^—Ag—P—C21	−33.48 (11)	P—C21—C26—C25	−176.9 (2)
Ag^i^—Ag—P—C21	19.73 (11)	C11—P—C31—C36	−28.3 (3)
P—Ag—N—C41	68.4 (3)	C21—P—C31—C36	82.4 (3)
I—Ag—N—C41	−155.9 (3)	Ag—P—C31—C36	−154.7 (3)
I^i^—Ag—N—C41	−39.6 (3)	C11—P—C31—C32	151.8 (2)
Ag^i^—Ag—N—C41	−95.5 (3)	C21—P—C31—C32	−97.5 (3)
P—Ag—N—C45	−102.6 (3)	Ag—P—C31—C32	25.4 (3)
I—Ag—N—C45	33.1 (3)	C36—C31—C32—C33	−0.3 (5)
I^i^—Ag—N—C45	149.5 (3)	P—C31—C32—C33	179.6 (3)
Ag^i^—Ag—N—C45	93.6 (3)	C31—C32—C33—C34	1.8 (6)
C31—P—C11—C12	169.8 (2)	C32—C33—C34—C35	−2.5 (6)
C21—P—C11—C12	60.2 (3)	C33—C34—C35—C36	1.7 (6)
Ag—P—C11—C12	−59.8 (2)	C32—C31—C36—C35	−0.5 (5)
C31—P—C11—C16	−68.2 (3)	P—C31—C36—C35	179.6 (3)
C21—P—C11—C16	−177.8 (2)	C34—C35—C36—C31	−0.2 (6)
Ag—P—C11—C16	62.2 (2)	C45—N—C41—C42	−0.1 (6)
C16—C11—C12—C13	55.0 (4)	Ag—N—C41—C42	−171.3 (3)
P—C11—C12—C13	176.8 (2)	N—C41—C42—C43	0.5 (6)
C11—C12—C13—C14	−55.7 (5)	C41—C42—C43—C44	0.0 (7)
C12—C13—C14—C15	55.5 (5)	C42—C43—C44—C45	−1.0 (7)
C13—C14—C15—C16	−54.9 (5)	C41—N—C45—C44	−1.0 (6)
C14—C15—C16—C11	55.2 (4)	Ag—N—C45—C44	170.5 (3)
C12—C11—C16—C15	−55.1 (4)	C43—C44—C45—N	1.5 (7)
P—C11—C16—C15	−177.2 (3)		

Symmetry codes: (i) −*x*+1, −*y*+1, −*z*+1.

### Table 2 Comparison of geometric parameters (Å, °) for selected [*X*Ag(py)(P_3_)_2_] (*X* = Cl, Br or I)

**Table:**

*X*	Ag—*X*	Ag—*X*	Ag···Ag	Ag—N	Ag—P	*X*—Ag—*X*	Ag—I—Ag
I^a^	2.8186 (4)	2.9449 (5)	3.1008 (6)	2.386 (3)	2.4436 (8)	114.947 (10)	65.053 (10)
I^b^	2.8402 (12)	2.8644 (8)	3.1130 (18)	2.392 (3)	2.4489 (12)	113.84 (4)	66.16 (4)
I^c^	2.814	2.875	3.343	2.422	2.440	108.02	71.98
Br^c^	2.701	2.733	3.499	2.391	2.415	99.85	80.15
Cl^c^	2.614	2.618	3.507	2.402	2.400	95.82	84.18

Notes: (a) This work; (b) Meijboom & Muller (2006); (c) Gotsis *et al.* (1989), extracted from the Cambridge Structural Database (Allen (2002), CSD CODES are VEFRUT for *X* = I, VEFRON for *X* = Br and VEFRIH for *X* = Cl.
